# Development of a scale for measuring orthosomnia: the Bergen Orthosomnia Scale (BOS)

**DOI:** 10.3389/frsle.2025.1640355

**Published:** 2025-10-14

**Authors:** Bodil V. Guldbrandsen, Kelly Baron, Øystein Vedaa, Bjørn Bjorvatn, Ståle Pallesen

**Affiliations:** ^1^Department of Psychosocial Science, University of Bergen, Bergen, Norway; ^2^Department of Family and Preventive Medicine, University of Utah, Salt Lake City, UT, United States; ^3^Department of Health Promotion, Norwegian Institute of Public Health (NIPH), Bergen, Norway; ^4^Department of Global Public Health and Primary Care, University of Bergen, Bergen, Norway; ^5^Norwegian Competence Center for Sleep Disorders, Haukeland University Hospital, Bergen, Norway

**Keywords:** orthosomnia, scale, perfect sleep, psychometrics, factor analysis, measurement invariance, interference, rigidity

## Abstract

**Introduction:**

This study aimed to extend the knowledge about orthosomnia, that is, excessive preoccupation with sleep, by developing a scale for its assessment.

**Methods:**

In Study 1, an initial item pool was presented to 34 sleep experts for assessment using the Delphi method. In Study 2, relevant items were administered to 994 survey respondents (mean age = 42 years, *SD* = 13.2) for exploratory and confirmatory factor analysis. Two factors were retained, reflecting “interference” and “rigidity,” each comprising six items. In Study 3, the scale was validated against multiple validated instruments reflecting sleep-related behaviors and perceptions, the five-factor personality traits, the dark triad personality traits, measures of obsessive-compulsive disorder (OCD) and health anxiety, as well as demographic variables, in a new sample (*n* = 473, mean age = 41 years, *SD* = 12.8).

**Results:**

The two-factor model demonstrated acceptable fit (root mean square of approximation = 0.07, comparative fit index = 0.96, Tucker–Lewis Index = 0.95) with Cronbach's alphas of 0.87 and 0.88, and 3-week test–retest reliability of 0.74 and 0.82, respectively. Both orthosomnia factors correlated positively with sleep effort, dysfunctional beliefs and attitudes about sleep, narcissism, perfectionism, OCD, and health anxiety. The interference factor correlated positively with insomnia, neuroticism, psychopathy, and Machiavellianism and negatively with conscientiousness. The rigidity factor correlated positively with conscientiousness.

**Conclusion:**

The new scale for assessing orthosomnia possesses good psychometric properties and provides clinicians and researchers with an instrument for further investigating this new sleep construct.

## 1 Introduction

The tendency to be excessively preoccupied with achieving perfect sleep has been referred to as orthosomnia (Baron et al., 2017). *Ortho* and *somnia* stem from Greek and literally mean “correct” and “sleep,” respectively. Although increased knowledge and focus on the importance of sleep are crucial, one cannot dismiss the possibility that for some, this may come with a cost in terms of sleep-related obsessions and anxiety (Baron et al., 2017; Van den Bulck, 2015). Little is known about the everyday behaviors of people with orthosomnia. A study of three cases described these individuals as preoccupied with tracking their sleep and feeling unrested during daytime (Baron et al., 2017). A few recent studies have been published in which orthosomnic tendencies were assessed, mainly by non-validated items (Kress et al., 2025). Others (Jahrami et al., 2024) have assessed orthosomnia with the Anxiety and Preoccupation about Sleep Questionnaire (APSQ). However, the APSQ is not intended to assess orthosomnia as such and is based on statements made by patients suffering from insomnia (Tang and Harvey, 2004). Another direction within this emerging field is to regard excessive preoccupation with sleep tracking as a defining aspect of orthosomnia (Jahrami et al., 2024; Khara and Kshatriya, 2025). In terms of sleep tracking, a parallel may be found in studies on excessive internet use, where differentiating between healthy and problematic use may be challenging (Schimmenti, 2023). So far, empirical evidence suggests the presence of both positive and negative effects of sleep tracking (Pallesen et al., 2025). Based on the current status of the field, the need for developing a scale with the specific aim of assessing orthosomnia is evident.

Several variables and traits can be assumed to be associated with orthosomia. Sleep effort, denoting the tendency to increase effort to sleep when facing sleep problems (Broomfield and Espie, 2005), could be relevant to orthosomnia as it is conceivable that individuals with a desire to achieve perfect sleep will increase their effort to fall asleep when facing sleep problems. Furthermore, as dysfunctional sleep-related beliefs and attitudes typically revolve around getting enough sleep and the impact of sleep disturbance on health and/or daytime functioning (Morin et al., 2007), it seems reasonable to expect that such beliefs and attitudes would be positively related to orthosomnia.

Previous research has described other instances of healthy behaviors expressed to the point of being unhealthy and obsessive, such as orthorexia (obsession with healthy eating; Bratman, 1997, 2000) and exercise addiction (Landolfi, 2013). Orthorexia and exercise addiction have been associated with both personality and several psychological conditions, which may also be relevant for orthosomnia. We therefore used orthorexia scales as a foundation for item generation for the orthosomnia scale described later in this article as the constructs share some core attributes (e.g., preoccupation with adhering to health behavior), often impairing quality of life (López-Gil et al., 2023; Baron et al., 2017). Due to their wording, items on orthorexia scales were deemed more relevant than those of exercise addiction scales. Hence, we emphasized orthorexia scales as a starting point for generating items for the orthosomnia scale. In terms of personality, individuals with orthorexia or exercise addiction have been found to have higher levels of neuroticism (McComb and Mills, 2019; Roncero et al., 2021; Strahler et al., 2020) and conscientiousness (Strahler et al., 2021) and lower levels of agreeableness (Costa and Oliva, 2012; Lichtenstein et al., 2014; Strahler et al., 2020). Exercise addiction is also positively associated with extraversion (Costa and Oliva, 2012; Lichtenstein et al., 2014; Metin et al., 2023). Furthermore, the Big Five personality traits have been associated with several sleep variables, making it plausible that personality traits could also be associated with orthosomnia. In particular, according to a meta-analysis, neuroticism is linked to poor sleep quality, whereas extraversion, conscientiousness, and agreeableness are weakly associated with good sleep quality (Guerreiro et al., 2024). Unfavorable traits like narcissism, psychopathy, and Machiavellianism, often referred to as the dark triad traits (Paulhus and Williams, 2002), have also been found to be positively associated with orthorexia and exercise addiction (Bruno et al., 2014; Cook et al., 2020; Miller and Mesagno, 2014; Oberle et al., 2017). The dark triad traits, in particular psychopathy and Machiavellianism, have also been associated with sleep disturbances and poorer sleep quality (Akram et al., 2018; Rahafar et al., 2022; Sabouri et al., 2016), possibly leading individuals who score high on these traits to strive for better sleep. Moreover, perfectionistic individuals are more likely to experience orthorexia (McComb and Mills, 2019) and exercise addiction (González-Hernández et al., 2023; Miller and Mesagno, 2014). Perfectionism has quite consistently been linked to sleep disturbance (Asgarabad et al., 2023; Johann et al., 2017). Additionally, orthorexia and exercise addiction have been associated with psychological conditions, such as obsessive-compulsive disorder (OCD) (Brytek-Matera et al., 2017; McComb and Mills, 2019; Meyer et al., 2021), and orthorexia has been associated with health anxiety (Barlow et al., 2024). Further studies suggest that OCD is associated with sleep impairment (Paterson et al., 2013). As orthosomnia might share some underlying psychological mechanisms with orthorexia and exercise addiction, one might expect some of the same personality traits and psychological conditions to be relevant in terms of orthosomnia.

Accordingly, in this study, we aimed to advance the knowledge about orthosomnia by developing a scale, the Bergen Orthosomnia Scale (BOS), that can be used to assess orthosomnia. In Study 1, we developed items for the scale using the Delphi method. Then, in Study 2, we assessed the factor structure and test–retest reliability of the new scale. Finally, in Study 3, we validated the scale against several convergent and divergent constructs theorized to be associated with orthosomnia and explored to what degree they are related to orthosomnia in multivariable analyses. The study was preregistered in Open Science Framework (https://doi.org/10.17605/OSF.IO/DP57K) before conducting analyses. The following hypotheses were posited: (1) The initial items to assess orthosomnia could be validated using the Delphi method, (2) the new orthosomnia scale will have acceptable psychometric properties (factor structure, test–retest reliability, and measurement invariance), and (3) the scores on the orthosomnia scale would correlate positively with known psychological scales assessing sleep variables (insomnia, sleep effort, and dysfunctional beliefs about sleep), different aspects of personality (neuroticism, conscientiousness, narcissism, psychopathy and Machiavellianism), and psychological conditions (perfectionism, obsessive-compulsive tendencies and health anxiety) previously associated with orthorexia or exercise addiction. Some traits (agreeableness, extraversion, and openness) were expected to be inversely related to orthosomnia.

## 2 Materials and methods

### 2.1. Samples and procedures

#### 2.1.1. Sample and procedure in Study 1

For Study 1, we reviewed scales assessing orthorexia, to select items for the BOS: Teruel Orthorexia Scale (TOS; Barrada and Roncero, 2018), ORTO-15 Questionnaire (Donini et al., 2005), and the Eating Habits Questionnaire-21 (EHQ-21; Gleaves et al., 2013). Items deemed relevant, based on a subjective evaluation made by the first and last author, were rewritten to refer to sleep and initially included, along with one item added about purchasing equipment to track sleep, resulting in 51 items. In line with the Delphi method, initial items were sent to 493 sleep experts by email, who were asked to rank each item from 1 (*should definitely not be part of the final assessment tool*) to 3 (*neutral*) to 5 (*should definitely be a part of the final assessment tool*). Sleep experts were operationalized as having published articles in *Sleep Health* or *Behavioral Sleep Medicine* in 2022 or 2023. They could propose rephrasing of items, additional items, and provide comments regarding the response alternatives. After feedback, a revised item list was sent out for a second assessment to the sleep experts participating in the first round. Items that 70% or more deemed relevant (*should be part of the final assessment tool* or *should definitely be part of the final assessment tool*) were kept for further investigation (Hasson et al., 2000). The Delphi study participants were 50% female and 50% male in the first round (*n* = 34), with a mean age of 46 years (*SD* = 11.9). In the second round, 44% females and 56% males (*n* = 18) participated, with a mean age of 48 years (*SD* = 10.6).

#### 2.1.2. Sample and procedure in Study 2

For Study 2, we used the online research platform Prolific (Palan and Schitter, 2018) to administer the proposed orthosomnia scale to 1,000 participants. Attention check items were added to exclude participants who responded carelessly (Brühlmann et al., 2020). Participants were paid an hourly rate of GBP 10 per hour. All participants were from the United Kingdom and had to be between 18 and 80 years of age, have a minimum of 95% approval rating on Prolific, and have completed a minimum of 10 surveys on Prolific. The final sample comprised 45% males, 53% females, and 1% other, with a mean age of 42 years (*SD* = 13.2; *n* = 994). The sample was randomly divided into two; one half for exploratory factor analysis (EFA; *n* = 497) and the other half for confirmatory factor analysis (CFA; *n* = 497), respectively. Three weeks later, a subsample of 300 participants (those who were first to respond) of the initial sample were reinvited to complete the orthosomnia scale to calculate the 3-week test–retest reliability. The sample for test–retest reliability consisted of 49% males, 49% females, and 1% other, with a mean age of 43 years (*SD* = 12.9; *n* = 297).

#### 2.1.3. Sample and procedure in Study 3

In terms of Study 3, the BOS, along with instruments measuring potential correlates of orthosomnia: insomnia symptoms, sleep effort, dysfunctional beliefs, and attitudes about sleep, health anxiety, symptoms of OCD, the five-factor personality traits, perfectionism, and the dark triad personality traits, was distributed to a new sample of 500 participants recruited from Prolific to investigate the convergent validity of the new scale. The same exclusion criteria (failing attention check items) were used as for Study 2. The sample consisted of 49% males, 50% females, and 0.4% other, with a mean age of 41 years (*SD* = 12.8; *n* = 473).

### 2.2. Instruments/questionnaires

#### 2.2.1. Instruments used in Study 1

The participants provided information about gender and age. The initial version of the BOS included 51 items, with response options on a 7-point Likert scale (1 = *Strongly disagree*, 2 = *Disagree*, 3 = *Somewhat disagree*, 4 = *Neither agree nor disagree*, 5 = *Somewhat agree*, 6 = *Agree*, and 7 = *Strongly agree*). In the second round of Study 1, some items were added, leaving the BOS with 54 items. After two rounds of the Delphi study (where items not deemed relevant were removed), the proposed BOS included 28 items (marked in bold in the list of all items; Appendix A).

#### 2.2.2. Instruments used in Study 2

Participants provided information about age, gender, level of education, current main employment status, personal income per year (after tax), marital status, and childcare responsibility. They further completed the 28-item version of the BOS. Sample demographics are presented in Appendix B.

#### 2.2.3. Instruments used in Study 3

Participants were asked to provide the same information as in study 2. The demographic variables were made dichotomous. For gender, the “other” category was omitted due to few responses. Regarding education, a distinction was made between not having completed higher education (1) and having completed any form of higher education (2), the latter defined as having completed technical/community college, undergraduate degree (BA/BSc/other), graduate degree (MA/MSc/MPhil/other), or doctorate degree (PhD/other). In terms of employment, a distinction was made between not working (1) and working/studying (2). A distinction was also made between making GBP 49,999 or less yearly (1) and GBP 50,000 or more yearly after tax. Furthermore, the child variable was dichotomized into not having children (1) and having children (2), and the partner variable was divided into not living with a partner (1) and living with a partner (2). The final BOS scale comprised the 12 items identified through factor analysis in Study 2. The 12 items are listed in Appendix C. The two factors reflected interference and rigidity, respectively.

##### 2.2.3.1. Mini international personality item pool

The Mini International Personality Item Pool (Mini-IPIP) measures the five-factor model (neuroticism, extraversion, intellect/imagination/openness, agreeableness, and conscientiousness) of personality (Donnellan et al., 2006). A higher score on each subscale indicates a higher tendency level of that trait. The internal consistency for the Mini-IPIP subscales was good, with Cronbach's α = 0.86 for extraversion, α = 0.85 for agreeableness, α = 0.71 for conscientiousness, α = 0.80 for neuroticism, and α = 0.80 for the intellect/imagination subscale.

##### 2.2.3.2. Short health anxiety inventory

The Short Health Anxiety Inventory (SHAI) measures health anxiety and fear of becoming ill (Salkovskis et al., 2002). Two factors correlated highly (*r* = 0.516, *p* < 0.001); hence, a composite score was used in the analysis. A higher SHAI score indicates higher levels of health anxiety (α = 0.92).

##### 2.2.3.3. Dysfunctional beliefs and attitudes about sleep-16

The Dysfunctional Beliefs and Attitudes about Sleep−16 (DBAS-16) measures to what extent one holds dysfunctional beliefs or attitudes about sleep (Morin et al., 2007). Higher scores indicate more dysfunctional beliefs or attitudes about sleep (α = 0.90).

##### 2.2.3.4. Glasgow sleep effort scale

This scale measures how much effort individuals exert to sleep (Broomfield and Espie, 2005). The Glasgow Sleep Effort Scale (GSES) items were scored so that higher GSES scores indicate more sleep effort (α = 0.84).

##### 2.2.3.5. Bergen insomnia scale

The Bergen Insomnia Scale (BIS) measures insomnia symptoms (Pallesen et al., 2008). Higher scores indicate more insomnia symptoms (α = 0.87).

##### 2.2.3.6. Obsessive-compulsive inventory-revised quiestionnaire

The Obsessive-Compulsive Inventory–Revised (OCI-R) questionnaire assesses symptoms of OCD (Foa et al., 2002), where higher scores indicate more OCD symptoms (α = 0.92).

##### 2.2.3.7. Frost multidimensional perfectionism scale

The Frost Multidimensional Perfectionism Scale (FMPS) was initially developed by Frost et al. (1990) and later revised by Stöber (1998). A total composite score was calculated, where higher scores indicate higher levels of perfectionism (α = 0.94).

##### 2.2.3.8. The dark triad dirty dozen

The Dark Triad Dirty Dozen (DTDD) assesses three unfavorable traits: narcissism, psychopathy, and Machiavellianism (Jonason and Webster, 2010). Higher scores on each subscale indicate higher levels of the trait. The internal consistency for the three subscales was good: α = 0.85, 0.86, and 0.77, respectively.

### 2.3 Statistical analysis

#### 2.3.1. Statistical analysis for Study 1

In Study 1, descriptive statistics (means, *SD*, and percentages) were calculated.

#### 2.3.2. Statistical analysis for Study 2

Suitability for EFA was assessed using the Kaiser–Meyer–Olkin measure of sampling adequacy and the Bartlett's test of sphericity. The 28 items were subjected to an EFA, with maximum likelihood (ML) extraction method and oblique (direct oblimin) rotation method, using SPSS version 28. The number of factors retained was decided based on the scree plot. In line with Costello and Osborne (2005), only factors with five or more item loadings ≥0.6 in the pattern matrix were kept to identify solid factors. The model identified through the EFA was further investigated through CFA, using SPSS Amos 28. The fit was evaluated by investigating chi-square, root mean square of approximation (RMSEA), comparative fit index (CFI), and the Tucker–Lewis Index (Hu and Bentler, 1999). Configural invariance was tested by investigating the fit of multigroup models for both age and gender groups (Byrne, 2016). Metric invariance and scalar invariance were investigated using ΔCFI, where the value should be < 0.01 to not reject the null hypothesis of invariance (Cheung and Rensvold, 2002). The age groups were established using a median split, resulting in groups of 18–39 and 40–80 years. Pearson product-moment correlation coefficient was used to investigate the test–retest reliability of the BOS, and Cronbach's alpha was calculated to measure its internal consistency.

#### 2.3.3. Statistical analysis for Study 3

A bivariate correlation analysis was used (significance level set to 0.001). Due to statistical overlap between the variables in the bivariate analyses, the contribution of each variable was further investigated using a standard linear multiple regression analysis. Preliminary analyses were conducted to ensure the assumptions of normality, linearity, multicollinearity, and homoscedasticity were not violated (Tabachnick and Fidell, 2014). The dependent variables in Study 3 comprised the two orthosomnia factors identified in Study 2. The demographic independent variables were age, gender, education, employment, income, and childcare responsibility. The additional independent variables comprised insomnia symptoms (BIS score); sleep effort (GSES score); dysfunctional beliefs and attitudes about sleep (DBAS-16 score); health anxiety (SHAI score); OCD-symptoms (OCI-R score); neuroticism, extraversion, agreeableness, and conscientiousness (Mini-IPIP); perfectionism (FMPS score); and narcissism, psychopathy, and Machiavellianism (DTDD subscale scores).

## 3 Results

### 3.1. Results for Study 1

Most of the 51 initial items were revised, and 7 more items were added after the feedback. A few items were removed, as they were nearly identical to other items. This resulted in 54 items after the first round of feedback. At least 70% of the experts deemed 26 items relevant, and they were thus included in the scale. Two more items were added based on feedback from one of the sleep experts (desire to track sleep). This resulted in a total of 28 items that were investigated further.

### 3.2. Results for Study 2

**Figure 1:**
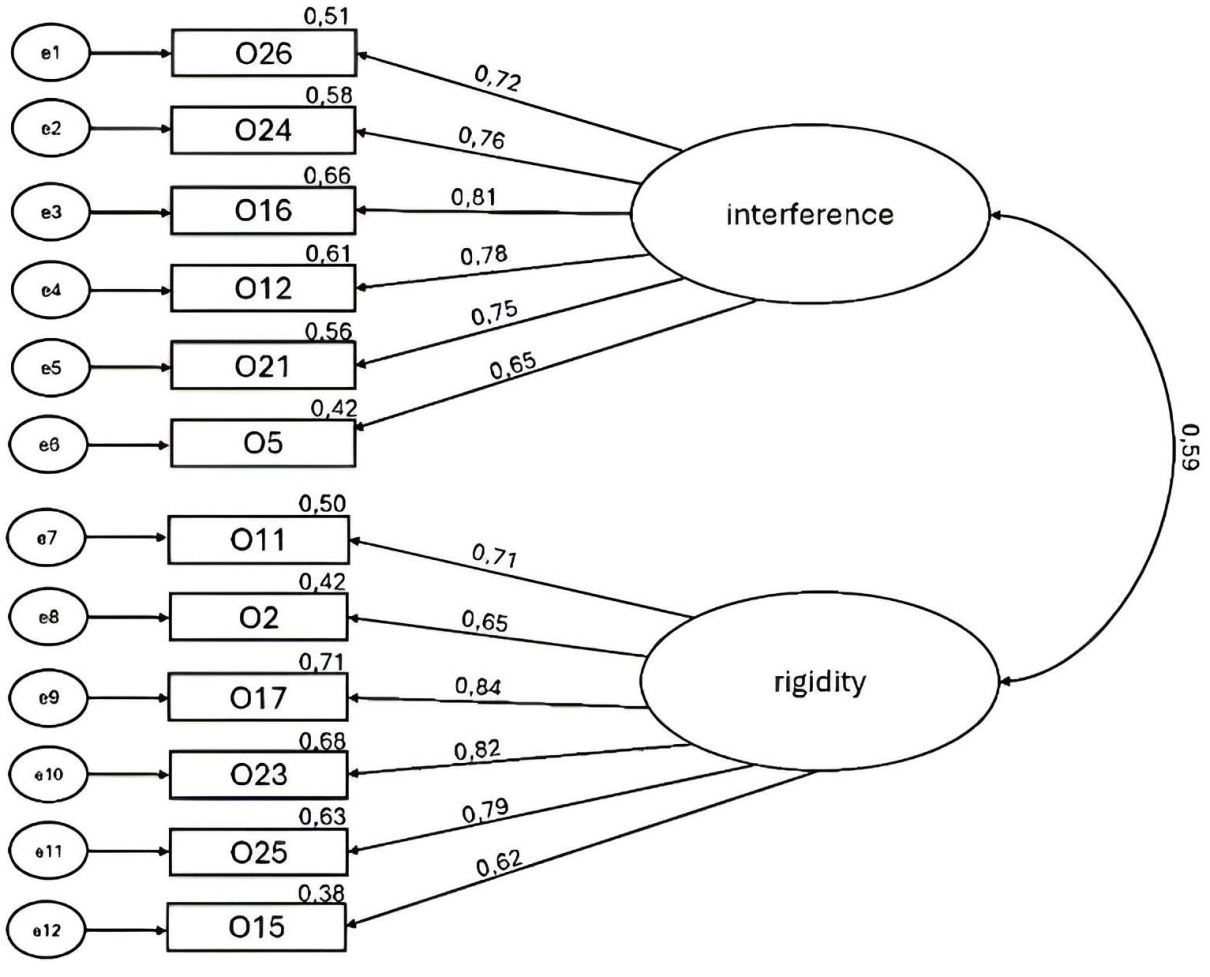
The model of Bergen Orthosomnia Scale depicting the 12 items distributed across the 2 factors, interference and rigidity, showing standardized factor loadings and squared multiple correlations (*n* = 497).

The EFA revealed five factors with eigenvalues exceeding 1, explaining 36%, 9%, 7%, 5%, and 4% of the variance, respectively. The scree plot was further inspected, revealing a break after the third factor, indicating that three factors should be kept for further investigation (Cattell, 1966). An investigation of the item loadings in the pattern matrix revealed six items loading ≥.6 on factor 1, two items loading ≥0.6 on factor 2, and six items loading ≥0.6 on factor 3. The interpretation of the factors revealed that factor 1 reflected preoccupation with sleep interfering with other aspects of life, factor 2 contained items about tracking sleep with devices, and factor 3 contained items measuring rigidity of sleep habits. As only two items loaded on factor 2, that factor was discarded. All factor loadings are presented in Table 1. The CFA confirmed the two-factor model revealed by the EFA. The factor structure is shown in Figure 1. The standardized factor loadings ranged from 0.62 (Item 15 on rigidity) to 0.84 (Item 17 on rigidity). All factor loadings were significant (*p* < 0.001). The two-factor model had an acceptable fit, χ^2^ (*df* = 53, *n* = 497) = 173.2, CFI = 0.96, RMSEA = 0.07 [90% CI [0.05, 0.08]], TLI = 0.95, *p* < 0.01. Configural invariance across gender, χ^2^ (*df* = 106, *n* = 497) = 221.66, CFI = 0.96, RMSEA = 0.05 [90% CI = [0.04, 0.06]], TLI = 0.95, and age, χ^2^ (*df* = 106, *n* = 497) = 222.46, CFI = 0.96, RMSEA = 0.05 [90% CI [0.04, 0.06]], TLI = 0.95, was shown. Evidence of metric and scalar invariance across gender (ΔCFI = 0.006 and ΔCFI = 0.009, respectively) and metric invariance across age (ΔCFI < 0.001) was found. After releasing the constraint of the intercepts on Items 11 and 12 on rigidity, the model reached partial scalar invariance across age groups (ΔCFI = 0.003). The internal consistency of the interference and rigidity subscales was good, with α = 0.87 and α = 0.88, respectively, and α = 0.90 for the whole scale (all 12 items). Test–retest reliability was acceptable, both for the interference (*r* = 0.77, *n* = 297, *p* < 0.001) and the rigidity (*r* = 0.82, *n* = 297, *p* < 0.001) subscale. The test–retest correlation coefficients for all items were satisfactory, ranging from 0.54 to 0.71 (see Table 2). The results for the three-factor solution (including the sleep tracking factor) are presented in Supplementary material showing that the two retained factors had a higher intercorrelation (*r* = 0.60) than they had with the sleep tracking factor (*r* = 0.33 and 0.36).

**Table 1:** Results from exploratory factor analysis of the orthosomnia scale.

**Item**	**Factor loading**		
	**1**	**2**	**3**
**Factor 1: Orthosomnia interference**			
26. My friends and family get annoyed by how much I talk about sleep.	**0.79**	−0.06	−0.12
24. In the past year, friends or family members have told me that I'm overly concerned with sleeping well.	**0.78**	−0.06	−0.06
16. My preoccupation with healthy sleep habits is a source of stress in my relationship(s).	**0.73**	−0.04	0.01
12. My healthy sleeping habits are restricting my life (e.g., making it difficult to go out with friends).	**0.73**	−0.01	0.03
21. I am preoccupied with sleep, even when I'm doing other things.	**0.69**	−0.06	0.03
5. My social relationships have been negatively impacted by my preoccupation with getting good quality sleep.	**0.61**	−0.04	0.05
**Factor 2: Measuring of sleep by a device**			
29. I think that tracking my sleep using a sleep app would help me sleep better.	0.07	**−0.91**	0.05
28. Tracking my sleep using a sleep app would help ensure that I get good quality sleep.	0.08	**−0.87**	0.06
**Factor 3: Orthosomnia rigidity**			
11. I do not allow myself to deviate from my sleep routine.	0.13	0.17	**0.72**
2. I spend a lot of time on my bedtime routine to ensure that I get a good night's sleep.	0.01	0.09	**0.69**
17. I follow rules for good sleep habits rigidly.	0.23	0.05	**0.68**
23. I go to great lengths to make sure I get a good night's sleep every night.	0.23	0.03	**0.65**
25. I try to follow perfect sleep habits.	0.23	0.01	**0.63**
15. I feel at peace with myself when I follow a strict sleep routine.	0.08	−0.09	**0.62**
**Items with no loadings** >**0.60**			
9. Thinking about getting good enough sleep is interfering with my ability to concentrate on other tasks.	0.60	−0.09	−0.01
6. My preoccupation with healthy sleeping takes up a lot of my time.	0.59	−0.05	0.25
27. I am a perfectionist when it comes to sleep.	0.50	−0.04	0.34
14. I turn down social offers that will interfere with my sleep habits.	0.46	0.11	0.35
22. I avoid going out late with others because of my sleep routine.	0.43	0.10	0.41
7. I worry that I might not get enough sleep.	0.23	−0.30	0.01
20. I am better informed than others about healthy sleep habits.	0.16	−0.03	0.50
19. I feel in control when I get enough good quality sleep.	−0.09	−0.12	0.49
10. I strongly believe that you need to sleep well to stay healthy.	−0.14	−0.05	0.47
1. I feel good about myself when I've made sure I get good quality sleep.	−0.20	−0.13	0.47
13. I set limits for things that could interfere with my sleep (e.g., socializing in the evening).	0.41	0.12	0.45
3. I purchase products designed to help me sleep better (such as bedding, sleep tracking devices, supplements, sound machines, etc.).	0.12	−0.15	0.43
8. I feel overwhelmed and/or disappointed if I do not get enough sleep or do not sleep well at night.	0.20	−0.13	0.30
4. I feel guilty when I don't get enough sleep/sleep well.	0.17	−0.18	0.29

*N* = 497. The extraction method was maximum likelihood with an oblique (direct oblimin) rotation. Factor loadings above 0.60 are in bold.

**Table 2:** Test–retest correlations for all items in the final orthosomnia scale.

**Item**	**Test–retest correlation**
2. I spend a lot of time on my bedtime routine to ensure that I get a good night's sleep.	0.70^**^
5. My social relationships have been negatively impacted by my preoccupation with getting good quality sleep.	0.59^**^
11. I do not allow myself to deviate from my sleep routine.	0.66^**^
12. My healthy sleeping habits are restricting my life (e.g., making it difficult to go out with friends).	0.61^**^
15. I feel at peace with myself when I follow a strict sleep routine.	0.65^**^
16. My preoccupation with healthy sleep habits is a source of stress in my relationship(s).	0.61^**^
17. I follow rules for good sleep habits rigidly.	0.71^**^
21. I am preoccupied with sleep, even when I'm doing other things.	0.63^**^
23. I go to great lengths to make sure I get a good night's sleep every night.	0.58^**^
24. In the past year, friends or family members have told me that I'm overly concerned with sleeping well.	0.57^**^
25. I try to follow perfect sleep habits.	0.68^**^
26. My friends and family get annoyed by how much I talk about sleep.	0.54^**^

Three-week test–retest correlations.

^**^p < 0.001.

### 3.3. Results for Study 3

Means and standard deviations for all study variables are presented in Table 3, whereas Table 4 displays correlations for all study variables with the two orthosomnia factors. The multiple linear regression analysis results for the interference factor indicated that the independent variables explained 36% of the variance (*R*^2^ = 0.36, *F*_21, 449_ = 12.17, *p* < 0.001). OCD symptoms had the highest association (*b* = 0.36, *p* < 0.001). For the rigidity factor, the analysis indicated that the independent variables explained 25% of the variance (*R*^2^ = 0.25, *F*_21, 449_ = 8.56, *p* < 0.001). Insomnia (*b* = −0.23, *p* < 0.001), dysfunctional beliefs about sleep (*b* = 0.36, *p* < 0.001), conscientiousness (*b* = 0.26, *p* < 0.001), and obsessive-compulsive symptoms (*b* = 0.28, *p* < 0.001) showed the highest associations (see Table 5).

**Table 3:** Means and standard deviations for all study variables.

**Variable**	**M**	**SD**	***n***
Orthosomnia interference score	12.5^a^	6.2	473
Orthosomnia rigidity score	19.9^b^	7.4	473
Insomnia symptoms	16.6	10.3	473
Sleep effort	11.5	3.2	473
Dysfunctional beliefs and attitudes about sleep	83.2	28.0	473
Neuroticism	11.3	3.8	473
Conscientiousness	14.6	3.1	473
Agreeableness	15.2	3.3	473
Openness (imagination/intellect)	14.4	3.4	473
Extraversion	10.6	4.0	473
Narcissism	9.0	3.6	473
Psychoticism	7.8	3.2	473
Machiavellianism	8.4	3.6	473
Perfectionism	78.7	19.9	473
Symptoms of obsessive-compulsive disorder	15.5	12.7	473
Health anxiety	18.6	8.8	473

^a^95 percentile = 25.00.

^b^95 percentile = 33.00.

**Table 4:** Correlations for study variables with orthosomnia interference and orthosomnia rigidity.

**Variable**	**Orthosomnia interference**	**Orthosomnia rigidity**
Age	**−0.12** ^*^	−0.03
Gender^a^	0.04	0.04
Education^b^	0.08	0.06
Employment^c^	0.12	0.10
Income^d^	0.00	−0.09
Marital status^e^	0.01	−0.00
Children in childcare^f^	−0.01	−0.04
Insomnia symptoms	**0.28** ^*^	−0.00
Sleep effort	**0.39** ^*^	**0.14** ^*^
Dysfunctional beliefs about sleep	**0.45** ^*^	**0.30** ^*^
Neuroticism	**0.27** ^*^	0.04
Conscientiousness	**−0.13** ^*^	**0.18** ^*^
Agreeableness	−0.08	0.04
Openness (imagination/intellect)	−0.07	−0.02
Extraversion	0.02	−0.01
Narcissism	**0.28** ^*^	**0.18** ^*^
Psychopathy	**0.13** ^*^	−0.02
Machiavellianism	**0.18** ^*^	0.07
Perfectionism	**0.35** ^*^	**0.21** ^*^
Symptoms of obsessive-compulsive disorder	**0.51** ^*^	**0.29** ^*^
Health anxiety	**0.32** ^*^	**0.16** ^*^

Significant correlations are marked in bold.

^*^p < 0.001.

^a^Gender: male = 1, female = 2.

^b^Education: No higher education = 1, higher education = 2.

^c^Employment: Unemployed = 1, employed/student = 2.

^d^Income: Less than GBP 49,999 yearly = 1, more than GBP 50,000 yearly = 2.

^e^Marital status: Living alone = 1, living with partner = 2.

^f^Children in childcare: No children = 1, one or more children in childcare = 2.

**Table 5:** Results from the multiple linear regression analysis, showing the contribution of each independent variable on the two orthosomnia factors.

**Variable**	**Orthosomnia interference**					**Orthosomnia rigidity**				
	***B***	***SE***	β	***t***	***p***	***B***	***SE***	β	***t***	***p***
Age	0.03	0.02	0.05	1.19	0.234	0.04	0.03	0.06	1.35	0.179
Gender^a^	0.60	0.55	0.05	1.10	0.274	0.59	0.68	0.04	0.87	0.386
Education^b^	0.68	0.54	0.05	1.25	0.213	0.43	0.68	0.03	0.63	0.528
Employment^c^	1.43	0.68	**0.09**	2.10	0.037	2.37	0.85	**0.12**	2.77	0.006
Income^d^	−0.13	0.76	−0.01	−0.18	0.860	−2.81	0.95	**−0.12**	−2.96	0.003
Marital status^e^	−0.28	0.53	−0.02	−0.53	0.595	−0.60	0.66	−0.04	−0.91	0.362
Children in childcare^f^	0.20	0.53	0.02	0.37	0.713	−0.36	0.66	−0.02	−0.55	0.583
Insomnia symptoms	−0.03	0.03	−0.05	−1.00	0.320	−0.16	0.04	**−0.23**	−4.07	< 0.001
Sleep effort	0.17	0.11	0.09	1.50	0.133	−0.02	0.14	−0.01	−0.13	0.899
Dysfunctional beliefs about sleep	0.06	0.01	**0.26**	4.76	< 0.001	0.10	0.02	**0.36**	6.32	< 0.001
Neuroticism	−0.05	0.09	−0.03	−0.59	0.554	−0.31	0.11	**−0.16**	−2.87	0.004
Conscientiousness	−0.02	0.08	−0.01	−0.25	0.802	0.59	0.11	**0.26**	5.69	< 0.001
Agreeableness	−0.29	0.10	**−0.16**	−2.85	0.005	−0.09	0.13	−0.04	−0.70	0.497
Openness (imagination/intellect)	−0.12	0.07	−0.07	−1.68	0.093	−0.09	0.09	−0.04	−1.04	0.301
Extraversion	0.18	0.07	**0.12**	2.57	0.011	−0.04	0.09	−0.02	−0.50	0.620
Narcissism	0.13	0.09	0.08	1.48	0.140	0.22	0.10	**0.11**	2.07	0.039
Psychopathy	−0.08	0.11	−0.04	−0.73	0.467	−0.16	0.14	−0.07	−1.14	0.253
Machiavellianism	−0.11	0.09	−0.07	−1.19	0.233	0.00	0.12	0.00	0.00	0.997
Perfectionism	0.02	0.01	0.07	1.44	0.152	0.02	0.02	0.05	0.97	0.332
Symptoms of obsessive-compulsive disorder	0.18	0.03	**0.35**	6.54	< 0.001	0.16	0.03	**0.28**	4.83	< 0.001
Health anxiety	−0.01	0.04	−0.01	−0.09	0.925	0.02	0.05	0.03	0.49	0.662

Statistically significant β-values < 0.05 are marked in bold.

^a^Gender: male = 1, female = 2.

^b^Education: No higher education = 1, higher education = 2.

^c^Employment: Unemployed = 1, employed/student = 2.

^d^Income: Less than GBP 50,000 yearly = 1, GBP 50,000 or more yearly = 2.

^e^Marital status: Living alone = 1, living with partner = 2.

^f^Children in childcare: No children = 1, one or more children in childcare = 2.

## 4 Discussion

The present study is the first large-scale study of the orthosomnia construct with the aim of developing a scale for its assessment. The Delphi method functioned well in terms of content validity and supported, as such, Hypothesis 1. The BOS comprised two factors, interference and rigidity, each composed of six items. The two factors correlated positively (*r* = 0.45), suggesting that they are related but still separate. The two-factor model was found to have an acceptable fit, with measurement invariance for sex and age groups, good internal consistency, and acceptable test–retest reliability, supporting Hypothesis 2.

In line with the bivariate correlation analysis, age was inversely related to the orthosomnia interference subscale, which might reflect that young people have fewer obligations than older ones (Suh, 2007). In the regression analysis, being employed was positively associated with both orthosomnia factors. This suggests that being employed increases the probability that sleep schedules interfere with other obligations and that employment may cause more fixed/rigid sleep routines (van Tienoven et al., 2014). According to the results from the bivariate correlation analysis, individuals who experienced a preoccupation with sleep that interferes with their lives and follow rigid sleep rules were more likely to put more effort into their sleep and have dysfunctional beliefs about sleep. As sleep effort and dysfunctional beliefs about sleep have been found to disrupt good sleep (Espie, 2002; Morin et al., 2007), they could play an important role in maintaining orthosomnia. Preoccupation with sleep was also positively associated with perfectionism, OCD, and health anxiety. The higher standards of perfectionistic individuals and a higher tendency for rumination and cognitive perseveration (Xie et al., 2019) could explain how preoccupation with sleep interferes with life. The obsessing and strict rules in OCD also share similarities with the interference and rigidity factors of orthosomnia, which could explain this association. Finally, because healthy sleeping is associated with several health benefits, it is conceivable that people with health anxiety are more prone to developing orthosomnia. The association between orthosomnia and perfectionism, OCD, and health anxiety coincides with the literature on orthorexia (Barlow et al., 2024; Brytek-Matera et al., 2017; McComb and Mills, 2019) and exercise addiction (González-Hernández et al., 2023; Lejoyeux et al., 2008; Miller and Mesagno, 2014; Meyer et al., 2021), indicating that preoccupation with healthy behaviors may be a risk factor for obsessive tendencies also in terms of sleep. These results are in line with Hypothesis 3. Individuals with insomnia were more likely to experience a preoccupation with sleep that interfered with their lives but not follow rigid sleep rules. People with insomnia typically worry about sleep during the day and generally use poor coping strategies (e.g., spending excessive time in bed) that may interfere with social interactions (Espie, 2002). These results partly support Hypothesis 3. Regarding personality, results differed for the two orthosomnia factors. Individuals high on neuroticism were more likely to have a preoccupation with sleep that interferes with their lives but not keep rigid sleep rules. This could be explained by such individuals' tendency to worry but not follow strict health behaviors (Tackett and Lahey, 2017; Ruiz-Palomino et al., 2020). In contrast, conscientious individuals were more likely to follow rigid sleep rules but not let their preoccupation with sleep interfere with their lives. The finding coincides with conscientious individuals' tendency to value organizing, planning, and high levels of self-control (Jackson and Roberts, 2017). A natural preference for following such rules might make them feel less restrictive. It should also be noted that high scores on conscientiousness have been found to be associated with tendencies such as obsessionality, perfectionism, rigidity, and slowness to respond (Nettle, 2006), which is consistent with our findings. Furthermore, agreeableness and extraversion were unrelated to orthosomnia. Narcissism was associated with both orthosomnia factors, while psychopathy and Machiavellianism were associated only with the interference factor. The tendency of narcissistic individuals to desire habits and behavior superior to others (Miller and Mesagno, 2014; Oberle et al., 2017) could possibly explain the keeping of rigid sleep rules, while the lacking empathy and ability to consider the needs of others might explain the association of narcissism, psychopathy, and Machiavellianism with the interference factor (Paulhus and Williams, 2002; Nogueira et al., 2019). These findings partly support Hypothesis 3. Although the two BOS subscales correlated as expected with many relevant constructs, the size of the correlations was small to moderate, suggesting that the new scale has adequate discriminative validity. In terms of the regression analyses, the findings regarding dysfunctional beliefs about sleep and obsessive-compulsive behaviors paralleled those from the bivariate correlation analysis, attesting to these two factors as strongly associated with orthosomnia. Insomnia was unrelated to the rigidity factor in the correlation analysis but turned out to have one of the strongest (negative) associations with the orthosomnia rigidity factor in the regression analysis. This finding runs tandem with studies showing that insomnia is associated with inconsistent sleep habits/behavior (Bei et al., 2016). The new orthosomnia scale was in the bivariate correlational analyses related to the various sleep, personality, and health variables as expected. The scale did not correlate with some constructs (agreeableness, extraversion, and openness) it was expected to have an inverse relationship with. The results overall seem to attest to the convergent validity of the new scale.

### 4.1 Strengths and limitations

The present study possesses several strengths, especially in terms of factor analyses, as well as reliability and validity analyses. The sample sizes were adequate, and validation was conducted against well-known instruments. The scale was based on adapting previous scales of orthorexia. A limitation in this regard is that the selection of items from orthorexia scales to be rewritten was based on a subjective evaluation made by the first and last author. Furthermore, it should be noted that an alternative would be to use items from established sleep scales to develop the BOS. However, we regarded scales assessing orthorexia and exercise addiction as a better starting point as these constructs specifically reflect “excessive and rigid healthy behaviors,” which tap into the core of orthosomnia. One could also argue that a better approach would have been to generate items through interviews with sleep experts. Still, it should be noted that not many sleep experts have extensive knowledge and experience with orthosomnia. Furthermore, the sleep experts who took part in the survey were encouraged to rate each of the suggested items and suggest additional items. Another possible limitation was that sleep experts were defined as having published articles in two sleep journals during the last 2 years. We did not, however, ask them about their knowledge about orthosomnia or their competence when it comes to scale construction, which we acknowledge as limitations of the present study. Furthermore, we found that a high number of experts (*n* = 493) had to be invited to reach a reasonable number of experts (*n* = 34; response rate: 6.9%) for the Delphi approach. In this regard, it should also be noted as a limitation that only 53% of the experts who took part in the first Delphi round also took part in the second. It should further be noted that the final model is largely at the mercy of the initial item pool; hence, other relevant items might have been omitted. Another limitation is that several hypotheses about correlates of orthosomnia were taken from the literature related to orthorexia and exercise addiction. Although the latter two constructs may share some aspects with orthosomnia (e.g., being overly concerned with health behavior), the constructs are also distinct in terms of the specific behaviors involved, which, to a certain extent, weakens the rationale for the hypotheses.

One factor identified in the EFA was not kept for CFA (tracking sleep by using a sleep app). Seen from an etymological perspective, orthosomnia does not imply being overly concerned about sleep tracking. It can further be argued that people can have an excessive focus on sleeping correctly without tracking their sleep with, for example, smart watches. Sleep tracking might also reflect a general technical interest and curiosity about sleep, and some report such use as positive for their sleep (Pallesen et al., 2025). The results from the three-factor solution showed that the two retained factors had a higher intercorrelation than they had with the sleep tracking factor, which may suggest that sleep tracking is rather tangentially related to orthosomnia. Still, in all cases where the two retained factors had similar and significant correlations with other constructs (sleep effort, dysfunctional beliefs and attitudes about sleep, narcissism, perfectionism, obsessive-compulsiveness, and health anxiety), the sleep tracking factor had similar and significant associations. Furthermore, the fit indexes for the two- and three-factor models were comparable. Hence, it cannot be ruled out that sleep tracking should be included as a dimension of orthosomnia. Thus, future studies should investigate the use of sleep apps related to orthosomnia. Another limitation was that participants were only recruited from the United Kingdom, and the ethnicity of the participants was not assessed, which limits the generalizability.

As the present study is the first large-scale empirical study of orthosomnia, future studies are needed to investigate orthosomnia in different clinical and cultural populations. Ideally, the scale should also have been validated against sleep–wake data (e.g., sleep diaries), as well as objective sleep recordings. This should be addressed in future studies. It will also be important to identify clinically meaningful cutoff values. In addition, future studies among populations treated for orthosomnia should consider using this scale to determine the ability of the BOS to detect clinical improvement as well as identify antecedents and consequences of orthosomnia over time. Future studies should also investigate, through randomized controlled trials, the effects of existing treatments (e.g., cognitive behavioral therapy for insomnia) on individuals with orthosomnia.

### 4.2 Conclusion

We constructed a 12-item orthosomnia scale (BOS) with two subscales (interference and rigidity). Overall, the BOS exhibited strong psychometric properties. Both orthosomnia factors were positively associated with sleep effort, dysfunctional beliefs and attitudes about sleep, narcissism, perfectionism, OCD, and health anxiety. The efforts made in this study serve as an important foundation for future research into the phenomenon of orthosomnia.

## Data Availability

The raw data supporting the conclusions of this article will be made available by the authors, without undue reservation.
