# Supplementary material for: Development of a scale for measuring orthosomnia: the Bergen Orthosomnia Scale (BOS)
Source: Front Sleep. 2025 Oct 14;4:1640355. doi: 10.3389/frsle.2025.1640355 (PMC12713983; doi:10.3389/frsle.2025.1640355)
Supplement: Supplementary file 1 [file Presentation_1.pdf]

Appendix A

Orthosomnia Items Through All Sub-studies

Item	% of experts rated as relevant
**1. I feel good about myself when I've made sure I get good quality sleep.**	**78**
**2. I spend a lot of time on my bedtime routine to ensure that I get a good night's sleep.**	**95***
**3. I purchase products designed to help me sleep better (such as bedding, sleep tracking devices, supplements, sound machines, etc.).**	**89**
4. I believe that my sleep is better than that of most people.	23
**5. I feel guilty when I don't get enough sleep/sleep well.**	**89**
**6. My social relationships have been negatively impacted by my preoccupation with getting good quality sleep.**	**95***
7. My interest in getting good sleep is an important part of the way I am.	67
**8. My preoccupation with healthy sleeping takes up a lot of my time.**	**100**
**9. I worry that I might not get enough sleep.**	**78**
10. I don't mind spending money on things that may make me sleep better.	56
**11. I feel overwhelmed and/or disappointed if I do not get enough sleep or do not sleep well at night.**	**83**

Item	% of experts rated as relevant
12. I try to convince other people to follow my healthy sleep habits.	50
13. If I don't get enough sleep, I punish myself.	50
**14. Thinking about getting good enough sleep is interfering with my ability to concentrate on other tasks.**	**72**
**15. I strongly believe that you need to sleep well to stay healthy.**	**72**
16. I worry about sleep for several hours a day.	67
**17. I do not allow myself to deviate from my sleep routine.**	**94***
18. I am a good person when I try to sleep well.	50
**19. My healthy sleeping habits are restricting my life (e.g. making it difficult to go out with friends).**	**89***
20. I believe that getting enough sleep and good quality sleep improves my appearance.	56
21. I prefer to sleep by myself in order to obtain enough sleep and good sleep.	61
22. I have healthier sleep habits than most people.	28
**23. I set limits for things that could interfere with my sleep (e.g. socializing in the evening).**	**77**
**24. I turn down social offers that will interfere with my sleep habits.**	**89**
**25. I feel at peace with myself when I follow a strict sleep routine.**	**78***
**26. My preoccupation with healthy sleep habits is a source of stress in my relationship(s).**	**83***
27. Sleeping well brings me fulfillment in my life.	39

Item	% of experts rated as relevant
28. I have made efforts to improve my sleep health over time.	40
29. My sleeping habits/desire to have good sleeping habits affect the type of employment I would take.	44
30. If I were to sleep at a hotel, I would have difficulty finding hotels that provide good enough sleep environments.	44
**31. I follow rules for good sleep habits rigidly.**	**100***
**32. I feel in control when I get enough good quality sleep.**	**72**
**33. I am better informed than others about healthy sleep habits.**	**72**
**34. I am preoccupied with sleep, even when I'm doing other things.**	**72***
35. I get a satisfaction from following a bunch of "sleep rules".	56
36. I daydream about sleeping even better than I do now.	12
37. I feel good about myself when I follow healthy sleep habits.	61
**38. I avoid going out late with others because of my sleep routine.**	**77**
39. My sleep routine has gotten more complex.	61
**40. I go to great lengths to make sure I get a good night's sleep every night.**	**95***
41. Most of my free time revolves around thinking about or following healthy sleep habits.	66
**42. In the past year, friends or family members have told me that I'm overly concerned with sleeping well.**	**94***
43. I am distracted by thoughts of sleep.	50
44. It's very important to me to have good sleep routines.	66
45. I go out less since I began committing to healthy sleep habits.	61

Item	% of experts rated as relevant
**46. I try to follow perfect sleep habits.**	**78***
47. I use a smart watch (or other such device) to track my sleep.	56
48. I feel frustrated on nights I struggle to sleep, because I follow perfect sleep habits.	66
49. Following healthy sleep habits gives me an edge over others.	44
**50. My friends and family get annoyed by how much I talk about sleep.**	**72***
51. It’s very important for me to get 8 hours of sleep or more every night.	66
52. My daily activities are easily negatively affected by a poor night’s sleep.	55
53. If I don’t sleep well, I know I’ll be irritable, depressed or anxious the next day.	55
**54. I am a perfectionist when it comes to sleep.**	**100**
**55. I think that tracking my sleep using a sleep app would help me sleep better.**	
**56. Tracking my sleep using a sleep app would help ensure that I get good quality sleep.**	

Note. Items distributed to participants in Study 2 are marked in bold. Items 55 and 56 were added after the final Delphi round and were not assessed by sleep experts. * Items were kept after factor analysis and were a part of the final orthosomnia scale.

Appendix B

Sample Demographic Characteristics for Study 2 and Study 3

Baseline characteristic	Study 2		Study 3	
	n	%	*n*	%
**Gender**				
Male	453	45.6	234	49.5
Female	532	53.5	237	50.1
Other	9	0.9	2	0.4
**Age**				
18–40 years	532	53.5	245	51.8
41–60 years	345	34.7	182	38.5
61–80 years	117	11.8	46	9.7
**Education**				
No higher education	288	29.0	140	29.6
Higher education	705	70.9	333	70.4
**Employment**				
Not working	174	29.0	83	17.5
Working/student	705	70.9	390	82.5
**Income**				
49,999 GDP or less	889	89.4	416	87.9
50,000 GDP or more	105	10.6	57	12.1
**Marital status**				
Living alone	368	36.8	183	38.7
Living with partner	628	63.2	290	61.3
**Childcare**				
No children in childcare	626	63.0	294	62.2
Children in childcare	368	37.0	179	37.8

Appendix C

Final 12 Orthosomnia Items

Item	Factor loading 1	2	3
**Factor 1: Orthosomnia interference**			
26. My friends and family get annoyed by how much I talk about sleep.	**.79**		
24. In the past year, friends or family members have told me that I'm overly concerned with sleeping well.	**.78**		
16. My preoccupation with healthy sleep habits is a source of stress in my relationship(s).	**.73**		
12. My healthy sleeping habits are restricting my life (e.g. making it difficult to go out with friends).	**.73**		
21. I am preoccupied with sleep, even when I'm doing other things.	**.69**		
5. My social relationships have been negatively impacted by my preoccupation with getting good quality sleep.	**.61**		
**Factor 3: Orthosomnia rigidity**			
11. I do not allow myself to deviate from my sleep routine.			**.72**
2. I spend a lot of time on my bedtime routine to ensure that I get a good night's sleep.			**.69**
17. I follow rules for good sleep habits rigidly.			**.68**
23. I go to great lengths to make sure I get a good night's sleep every night.			**.65**
25. I try to follow perfect sleep habits.			**.63**
15. I feel at peace with myself when I follow a strict sleep routine.			**.62**
