# Supplementary material for: Development of a scale for measuring orthosomnia: the Bergen Orthosomnia Scale (BOS)
Source: Front Sleep. 2025 Oct 14;4:1640355. doi: 10.3389/frsle.2025.1640355 (PMC12713983; doi:10.3389/frsle.2025.1640355)
Supplement: Supplementary file 2 [file Table_1.docx]

**Results**

The CFA supported the three-factor model revealed by the EFA. The factor structure is shown in Figure 1A. The standardized factor loadings ranged from 0.63 (item 15 on rigidity) to 0.98 (item 28 on sleep tracking). All factor loadings were significant (*p <* .001). The three-factor model had an acceptable fit with the data, χ^2^ (df = 74, *n* = 497) = 278.3, CFI = .947, RMSEA = .075 (90% CI = .065–.084), TLI = .935. The CFA further revealed configural invariance across gender; χ^2^ (df = 148, *n* = 493) = 358.15, CFI = .946, RMSEA = .054 (90% CI = .047–.061), TLI = .933, and across age; χ^2^ (df = 148, *n* = 497) = 360.85, CFI = .944, RMSEA = .054 (90% CI = .047–.061), TLI = .931. The analysis further provided evidence of metric and scalar invariance across gender (ΔCFI = .003 and ΔCFI = .004, respectively) and evidence of metric and scalar invariance across age (ΔCFI = .005 for both).

**Figure 1A**

**Factor Structure of the 3-Factor Orthosomnia Solution**

**
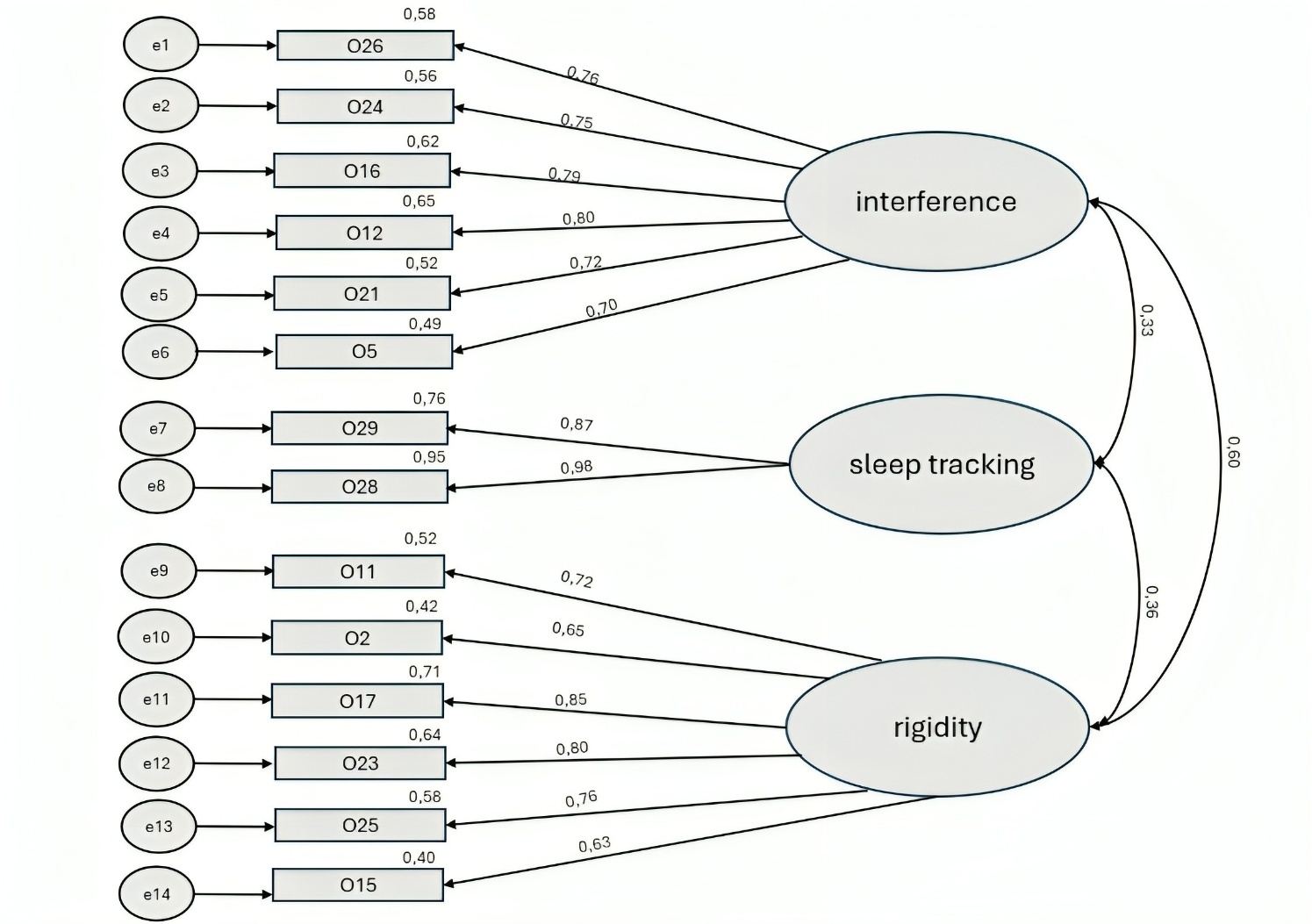
**

**Table 1A**

*Means and Standard Deviation for the Orthosomnia Sleep Tracking Subscale*

|  | M | SD | n |
| --- | --- | --- | --- |
| Orthosomnia sleep tracking scale | 6.6^1^ | 2.3 | 473 |

**Table 2A**

*Test-Retest Correlations for the Items in the Orthosomnia Sleep Tracking Subscale*

| **Item** | Test-retest correlation |
| --- | --- |
| 28. Tracking my sleep using a sleep app would help ensure that I get good quality sleep. | .70 |
| 29. I think that tracking my sleep using a sleep app would help me sleep better. | .74 |
| Composite score | .76 |

**Table 3A**

*Correlations for Study Variables with the Orthosomnia Sleep Tracking Subscale*

|  | Orthosomnia interference |
| --- | --- |
| Age | **-.27*** |
| Gender^a^ | -.07 |
| Education^b^ | .06 |
| Employment^c^ | **.17*** |
| Income^d^ | .03 |
| Marital status^e^ | -.01 |
| Children in childcare^f^ | -.06 |
| Insomnia symptoms | **.14*** |

| Sleep effort | **.24*** |
| --- | --- |
| Dysfunctional beliefs about sleep | **.28*** |
| Neuroticism | .10 |
| Conscientiousness | -.04 |
| Agreeableness | .06 |
| Openness (imagination/intellect) | .06 |
| Extraversion | .01 |
| Narcissism | **.21*** |
| Psychopathy | .06 |
| Machiavellianism | .11 |
| Perfectionism | **.19*** |
| Symptoms of obsessive-compulsive disorder | **.28*** |
| Health anxiety | **.20*** |

*Note*. Significant correlations are marked in bold, * *p <* .001. ^a^Gender: male = 1, female = 2, ^b^Education: No higher education = 1, higher education = 2, ^c^Employment: Unemployed = 1, employed/student = 2, ^d^Income: Less than 49,999 GBP yearly = 1, more than 50,000 GBP yearly = 2, ^e^Marital status: Living alone = 1, living with partner = 2, ^f^Children in childcare: No children = 1, one or more children in childcare = 2.

***(.60)Table 4A.***

*Results from the Multiple Linear Regression Analysis, Showing the Contribution of Each Independent Variable on the Orthosomnia Sleep Tracking Subscale*

|  | *B* | *SE* | β | *t* | *p* |
| --- | --- | --- | --- | --- | --- |
| Age | -.05 | .01 | **-.21** | -4.29 | <.001 |
| Gender^a^ | -.60 | .32 | -.09 | -1.91 | .057 |
| Education^b^ | -.09 | .31 | -.01 | -0.29 | .774 |
| Employment^c^ | .79 | .39 | **.09** | 2.00 | .046 |
| Income^d^ | .31 | .44 | .03 | 0.72 | .474 |
| Marital status^e^ | .13 | .31 | .02 | 0.43 | .670 |
| Children in childcare^f^ | -.53 | .31 | -.08 | -1.72 | .086 |
| Insomnia symptoms | .01 | .02 | .02 | 0.32 | .748 |
| Sleep effort | -.01 | .06 | -.01 | -0.08 | .938 |
| Dysfunctional beliefs about sleep | .02 | .01 | **.19** | 3.19 | .002 |
| Neuroticism | -.07 | .05 | -.08 | -1.34 | .180 |
| Conscientiousness | .08 | .05 | .08 | 1.72 | .087 |
| Agreeableness | .13 | .06 | **.13** | 2.17 | .030 |
| Openness (imagination/intellect) | .03 | .04 | .03 | 0.66 | .511 |
| Extraversion | -.00 | .04 | -.00 | -0.06 | .950 |
| Narcissism | .10 | .05 | .11 | 1.96 | .050 |
| Psychopathy | .07 | .07 | .07 | 1.02 | .310 |
| Machiavellianism | -.05 | .05 | -.05 | -0.86 | .390 |
| Perfectionism | .00 | .01 | .00 | 0.06 | .949 |
| Symptoms of obsessive-compulsive disorder | .03 | .02 | **.12** | 1.97 | .049 |
| Health anxiety | .02 | .02 | .06 | 0.98 | .326 |

*Note. B* = unstandardized regression coefficient, β = standardized regression coefficient. Statistically significant β-values < .05 are marked in bold. ^a^Gender: male = 1, female = 2, ^b^Education: No higher education = 1, higher education = 2, ^c^Employment: Unemployed = 1, employed/student = 2, ^d^Income: Less than 50,000 GBP yearly = 1, 50,000 or more GBP yearly = 2, ^e^Marital status: Living alone = 1, living with partner = 2, ^f^Children in childcare: No children = 1, one or more children in childcare = 2.

F(21,449) = 5.41, p<.01, R^2^=.20
